# Uric Acid as a Biomarker for Mood Disorders: A Comparative Study of Blood Uric Acid Levels Correlating With the Symptom Severity and Treatment Response

**DOI:** 10.7759/cureus.66784

**Published:** 2024-08-13

**Authors:** Sylviah Immanuel, Aruna Kaki, Ramya Rachel Jetty, Sudha Manaswini Vupputuri, Ramireddy K V, Arul Saravanan R

**Affiliations:** 1 Psychiatry, SRM Medical College Hospital and Research Center, Chennai, IND; 2 Psychiatry, District Hospital, Parvathipuram, IND; 3 Psychiatry, Andhra Medical College, Visakhapatnam, IND

**Keywords:** treatment response, symptom severity, mania, mdd, bpad, mood disorders, uric acid levels

## Abstract

Background

Bipolar affective disorder (BPAD) and major depressive disorder (MDD) are two mood disorders whose pathophysiology may involve the purinergic system. Elevated uric acid levels, associated with this system, can impact various behaviors in individuals affected by these conditions. In addition to genetic predisposition, blood uric acid levels can be impacted by various factors, including metabolic syndrome, the consumption of psychoactive medications, and other underlying kidney conditions such as gout.

Objective

The study aims to investigate the relationship between blood uric acid levels and mental health conditions, specifically BPAD subtypes (manic and depressive) and MDD. The study also examines changes in blood uric acid levels following treatment and evaluates the effectiveness of different treatment approaches in reducing uric acid levels.

Methodology

To be eligible to participate, individuals must have a confirmed diagnosis of BPAD (manic or depressive type) or MDD, according to the International Classification of Diseases (ICD-10). Blood uric acid levels were measured at both baseline and follow-up assessments. Symptoms were assessed weekly using standardized rating scales (Young Mania Rating Scale (YMRS) and Hamilton Rating Scale for Depression (HAM-D)) until treatment response was achieved, which was defined as a 50% reduction in initial scores on both scales. We used ANOVA to examine the differences among the three patient groups and paired sample t-tests to examine the changes in means before and after treatment conditions.

Results

A significant positive correlation was found between the severity of illness and serum uric acid levels across all three patient groups: those with BPAD-mania, BPAD-depression, and MDD. Notably, patients with BPAD-mania patients had significantly higher serum uric acid levels (5.2±0.9 mg/dL) compared to those with BPAD-depression (4.8±1.0 mg/dL) and MDD (4.0±1.1 mg/dL). After treatment, all patient groups exhibited a decrease in serum uric acid levels. The reduction in serum uric acid levels was pronounced in all patient groups, with decreases of 3.1±0.8 mg/dL in patients with BPAD-mania, 3.1±0.9 mg/dL in those with BPAD-depression, and 3.5±1.1 mg/dL in those with MDD. The study showed that the reduction in serum uric acid levels was significantly correlated with the severity of illness in patients with BPAD-mania, but not in those with BPAD-depression or MDD. Furthermore, the study found that treatment with lithium carbonate, sodium valproate, or carbamazepine was equally effective in reducing serum uric acid levels, regardless of the mood stabilizer used.

Conclusion

The study supports that dysfunction in the purine system might play a significant role in the development and progression of BPAD, suggesting that this phenomenon is not solely due to chronicity or medication exposure. This study also introduces a fresh perspective on the underlying biological processes that contribute to the development of BPAD and also sheds light on new treatment regimens targeting uric acid reduction in treating patients with bipolar disorder.

## Introduction

Mood disorders are classified into two primary categories: bipolar affective disorder (BPAD) and major depressive disorder (MDD) [1,2]. BPAD is characterized by recurring episodes of mania or depression, while MDD is marked by a persistent and pervasive feeling of sadness that lasts for at least two weeks, significantly impacting daily life and overall well-being [3]. Research has revealed that the purinergic system may play a vital role in the underlying mechanisms of BPAD and MDD [4-10].

The purinergic system is a crucial neurotransmitter pathway that ultimately leads to the production of uric acid, which has been implicated in the etiology and progression of various mental health disorders [11]. Uric acid can impact both pre- and postsynaptic neurons, as well as specific receptors on glial cell membranes [12]. This can affect the activity of various neurotransmitters, such as dopamine, gamma-aminobutyric acid, glutamate, and serotonin, all of which play an important role in the pathophysiology of mood disorders [13].

Recent studies found that individuals with BPAD have elevated uric acid levels compared to those without mental health issues and those with other mental health disorders [4,5]. Other studies suggest that uric acid levels may be a useful biomarker for distinguishing BPAD from MDD, particularly during manic episodes [6-9]. However, another study failed to find significant differences in uric acid levels between BPAD and MDD [10].

Serum uric acid levels can be influenced by various factors, including metabolic syndrome, substance abuse, kidney disorders, gout, and medication use [14,15]. For example, a meta-analysis study revealed that approximately 37% of individuals with BPAD also have metabolic syndrome, which is often associated with elevated uric acid levels [14]. Given the potential impact of these factors on uric acid levels, it is crucial to carefully select study samples to ensure the validity of the research. Moreover, there is a notable lack of literature examining the relationship between blood uric acid levels and clinical variables, as well as changes in blood uric acid levels following treatment in patients with BPAD-manic, BPAD-depressive, and MDD. Hence, the current study aims to fill this knowledge gap.

Building upon the existing literature, the current study aims to investigate the relationship between blood uric acid levels and clinical variables in patients with BPAD-mania, BPAD-depression, and MDD, as well as to examine the changes in blood uric acid levels following treatment in these patients’ populations.

## Materials and methods

**Figure 1:**
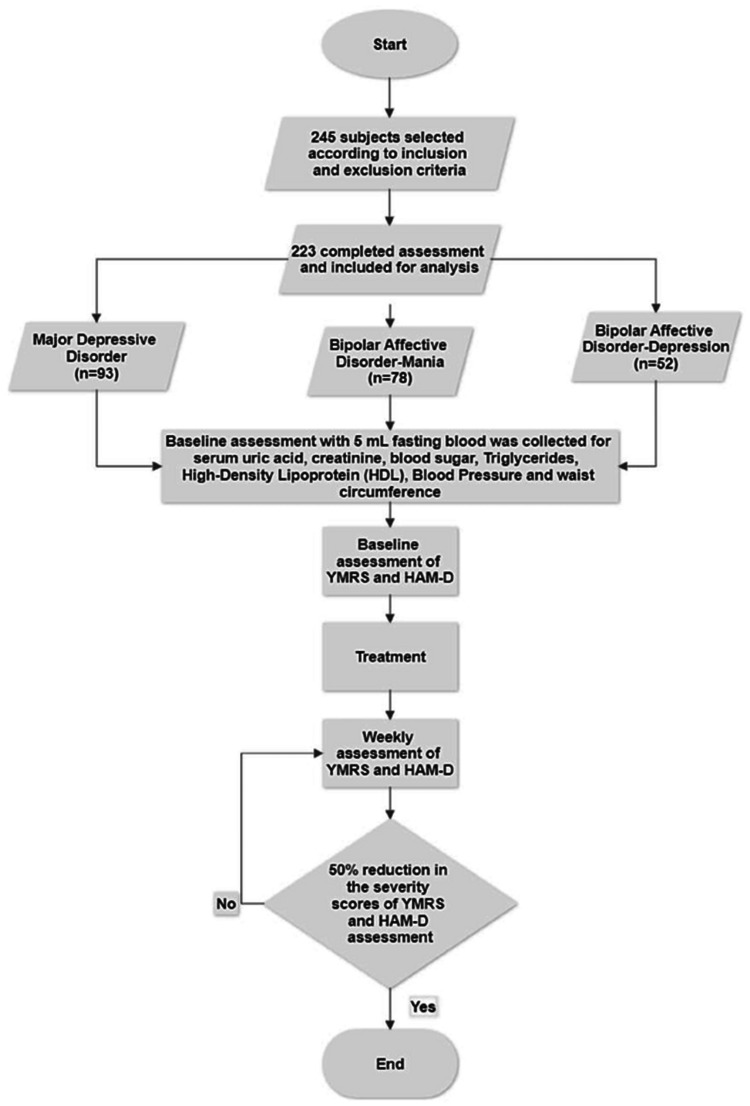
Representation of study flow diagram.

A visual representation of the study's flow chart is provided in Figure 1, illustrating the methodology and structure of the research. Recruitment for the study was conducted at a tertiary care psychiatry hospital in Visakhapatnam. The research employed a prospective study design, which spanned from September 1, 2019 to August 31, 2020.

A total of 223 adult patients aged 18-55 years were included in the study. Prior to the initiation of the study, the Institutional Scientific Committee and Ethics Committee approval was obtained, and Written Informed Consent was obtained from caregivers of all the participating BPAD-mania, BPAD-depression, and MDD by qualified psychiatrists. To be eligible for participation, individuals must have a confirmed diagnosis of BPAD-mania, BPAD-depression, and MDD as outlined in ICD-10 [16]. The individuals were receiving the appropriate treatment with antipsychotics, benzodiazepines, mood stabilizers, and antidepressants on a need basis. Individuals who fail to provide valid written consent will be excluded; please refer to Figure 5 in the Appendices for details. Excluded are individuals with other psychiatric and medical comorbidities. Additionally, individuals with renal disease, gout, or other conditions that impact serum uric acid levels are excluded. Individuals taking diuretics, probenecid, or sulfinpyrazone are also excluded due to their interaction with serum uric acid levels. Finally, individuals with a history of substance use disorder or those who have used alcohol or other psychoactive substances within one month will be excluded.

Upon admission, each participant was required to provide a 5-milliliter fasting blood sample, which was analyzed to determine the levels of serum uric acid, creatinine, fasting glucose, triglycerides, and high-density lipoprotein (HDL) cholesterol as represented in Figure 1. Additionally, waist circumference and blood pressure were measured. On the same day, patients' symptoms were evaluated using a standardized assessment tool. For patients with bipolar mania, Young Mania Rating Scale (YMRS) was used to assess the severity of symptoms. For patients with bipolar depression or MDD, the Hamilton Depression Rating Scale (HAM-D) was employed. The symptoms were re-assessed on a weekly basis until a treatment response was evident, defined as a 50% reduction in the initial scores on the YMRS and HAM-D scales. Serum uric acid levels were also re-assessed on the same day that treatment response was attained.

The self-designed form (refer to Figures 6, 7 in Appendices) collects personal and socio-demographic data from study participants, including identification details such as name, age, place of origin, marital status, religion, education, occupation, and income.

The YMRS, originally developed by Young et al. in 1978 [17], is a widely employed and well-established rating scale used to evaluate and assess the severity of manic symptoms. The YMRS is comprised of 11 items (refer to Figure 8 in Appendices), with a scoring system that varies across categories. Four items are assessed on a scale of 0-8 to measure the intensity of symptoms, including irritability, speech disturbances, thought content abnormalities, and disruptive or aggressive behavior. The remaining seven items assessing the severity are scored on a scale of 0-4.

The HDRS, originally developed by Max Hamilton in 1960, is a widely utilized tool for assessing the severity of depression [18]. This scale consists of 17 items (refer to Figures 9, 10 in Appendices), which are scored as follows: nine items with a score range of 0 to 4, seven items with a score range of 0 to 2, and one item with a score range of 0 to 3.

The assessment involves measuring the reduction of chromogen (such as tungstate) by uric acid, resulting in a color change that can be quantified. This is achieved through the enzymatic determination of uric acid through oxidation by uricase. Uricase converts uric acid into allantoin through an enzymatic reaction.

The study's results were analyzed using Minitab statistical software, where both ANOVA and paired-sample t-tests were applied to examine the data. ANOVA was employed to identify whether there were significant differences in the means among the three groups: individuals with symptoms of BPAD-mania, those with BPAD-depression, and those with MDD. As a powerful inferential statistical tool, ANOVA enabled us to uncover patterns in the data and understand relationships between variables. Additionally, a paired-sample t-test was used to analyze the means of two related groups, such as before-and-after treatment, providing valuable insights into treatment outcomes. It is to be noted that in this study, we considered results statistically significant if P < 0.05.

## Results

Socio-demographic profile

A total of 245 individuals who were diagnosed with BPAD-mania/BPAD-depression/MDD participated in this study. After the initial sampling process, 22 subjects were lost to follow-up, leaving a remaining sample of 223 participants that comprised the final study population. The diagnostic breakdown of the sample was as follows: 41.7% (93 participants) were diagnosed with MDD, 35.0% (78 participants) were diagnosed with BPAD-mania, and 23.3% (52 participants) were diagnosed with BPAD-depression.

The socio-demographic characteristics of all three groups are represented in Table 1. The mean age of participants in each group was 38.1 years (with a standard deviation of 10.4) for the BPAD-mania group, 38.0 years (with a standard deviation of 9.4) for the BPAD-depression group, and 36.6 years (with a standard deviation of 9.3) for the MDD group. Across all groups, males comprised the majority of participants, and more than 60% of subjects were married and unemployed.

**Table 1:** Socio-demographic profiles of all subjects. MDD - major depressive disorder

		BPAD-mania		BPAD-depression		MDD	
		N	%	N	%	N	%
Total subjects		78	34.7	52	23.1	93	58.7
Age, years	Mean	38.1		38		36.6	
	SD	10.4		9.4		9.3	
Gender	Male	42	53.8	32	61.5	48	51.6
	Female	36	46.2	20	38.5	45	48.4
Literacy	Literates	43	55.1	35	67.3	67	72.0
	Illiterates	35	44.9	17	32.7	26	28.0
Marital status	Married	51	65.4	33	63.5	57	61.3
	Unmarried	15	19.2	14	26.9	23	24.7
	Others	12	15.4	5	9.6	13	14.0
Employment	Employed	30	38.5	18	34.6	35	37.6
	Unemployed	48	61.5	34	65.4	58	62.4

Clinical variables

Table 2 presents the clinical variables for all three groups. The mean duration of illness was found to be 9.7±8.0 years for patients with BPAD-mania, 3.6±2.5 years for those with BPAD-depression, and 3.5±2.3 years for individuals with MDD (Table 2). A significant and positive association was found between the length of illness and serum uric acid levels across all groups, suggesting that as the duration of illness increases, serum uric acid levels also tend to rise. A significant correlation was noted between age and blood uric acid levels in all three groups, suggesting that with age blood uric acid levels tend to rise.

The serum uric acid levels in the three groups exhibited a statistically significant difference before treatment. The mean levels of uric acid levels in the BPAD-mania group were 5.2±0.9 mg/dL. In contrast, the mean levels in the BPAD-depression and MDD groups were 4.8±1.0 mg/dL and 4.0±1.1 mg/dL, respectively (Table 2). After treatment, the mean serum uric acid levels decreased to 3.1±0.8 mg/dL in the BPAD-mania group, 3.1±0.9 mg/dL in the BPAD-depression group, and 3.5±1.1 mg/dL in the MDD group (Table 2). ANOVA revealed a statistically significant difference in serum uric acid levels across the three groups, both at the pre-treatment and post-treatment time points, suggesting that there were meaningful changes in uric acid levels over time. Additionally, the results based on the paired t-test showed a statistically significant decrease in serum uric acid levels across all three treatment groups, indicating a significant improvement in uric acid levels following treatment when compared to baseline levels (Table 2).

**Table 2:** Comparison of clinical variables for all three groups. HAM-D - Hamilton Rating Scale for Depression, BPAD - Bipolar affective disorder, YMRS - Young Mania Rating Scale, MDD - major depressive disorder

Type of illness			BPAD-mania	BPAD-depression	MDD
Duration of illness		N	78	52	93
		Mean	9.73	3.56	3.52
		SD	7.99	2.51	2.3
		P-value	0.001*		
Serum uric acid levels, mg/dL	Before treatment	N	78	52	93
		Mean	5.23	4.69	3.95
		SD	0.91	0.99	1.08
	After treatment	N	78	52	93
		Mean	3.14	3.05	3.45
		SD	0.76	0.87	1.02
		P-value	0.01*	0.02*	0.04*
YMRS score	Before treatment	N	78		
		Mean	34.06		
		SD	8.87		
	After treatment	N	78		
		Mean	15.97		
		SD	4.51		
		P-value	0.01*		
HAM-D score	Before Treatment	N		52	93
		Mean		18.9	18.49
		SD		4.28	4.89
	After Treatment	N		52	93
		Mean		9.02	8.87
		SD		2	2.37
		P-value		0.000*	0.000*

The YMRS scores for subjects with BPAD showed a significant improvement after treatment. Specifically, the mean YMRS score decreased from 34.1 ± 8.9 points before treatment to 16.0 ± 4.5 points after treatment (Table 2). In addition, the HAM-D scores for patients with BPAD-depression and MDD also improved significantly following treatment. The mean HAM-D score for BPAD-depression decreased from 18.9 ± 4.3 points before treatment to 9.1 ± 2.0 points after treatment (Table 2), while the mean HAM-D score for MDD decreased from 18.5 ± 4.9 points to 8.9 ± 2.4 points (Table 2).

**Figure 2:**
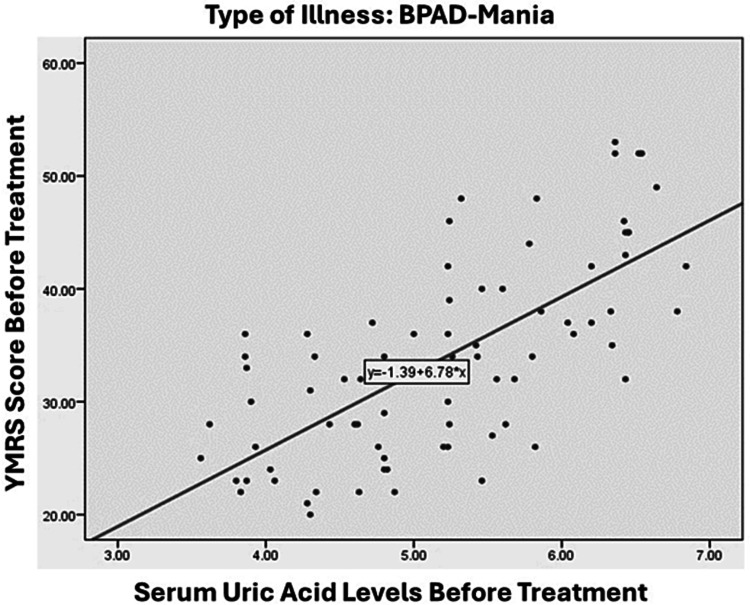
Correlation between YMRS score and blood uric acid level in patients with BPAD-mania. YMRS - Young Mania Rating Scale, BPAD - Bipolar affective disorder

**Figure 3:**
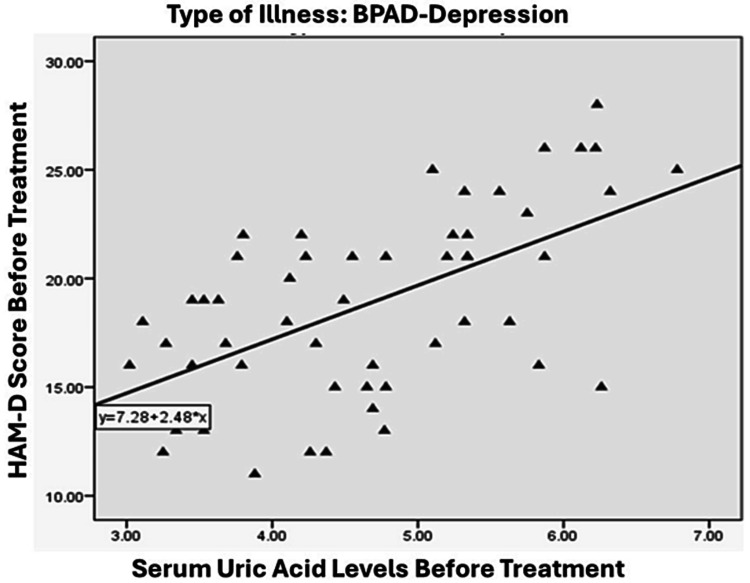
Correlation between HAM-D score and blood uric acid level in patients with BPAD-depression. HAM-D - Hamilton Rating Scale for Depression, BPAD - Bipolar affective disorder

**Figure 4:**
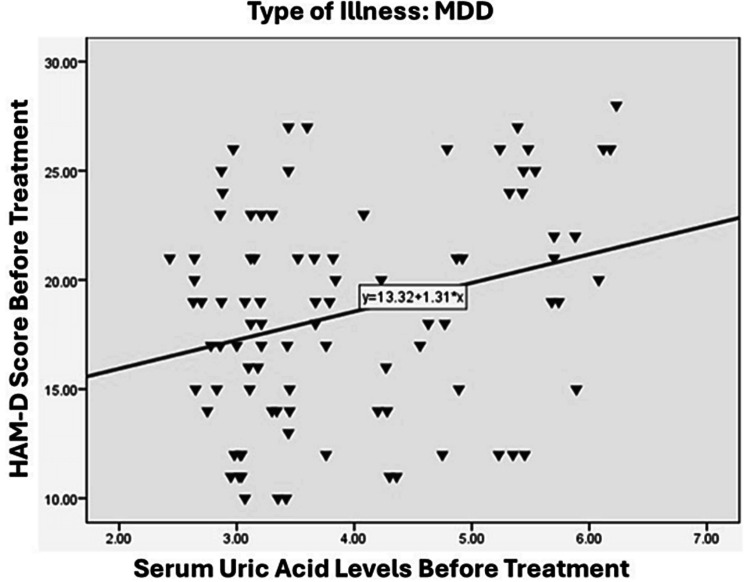
Correlation between HAM-D score and blood uric acid level in MDD. HAM-D - Hamilton Rating Scale for Depression, MDD - major depressive disorder

**Figure 5:**
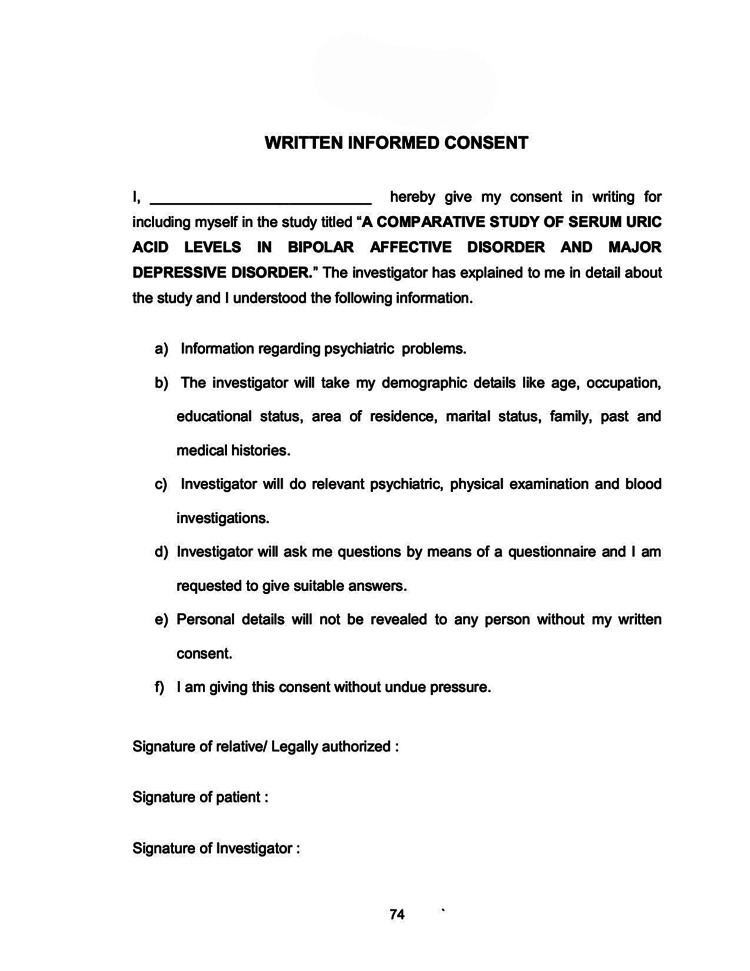
Written informed consent form.

**Figure 6:**
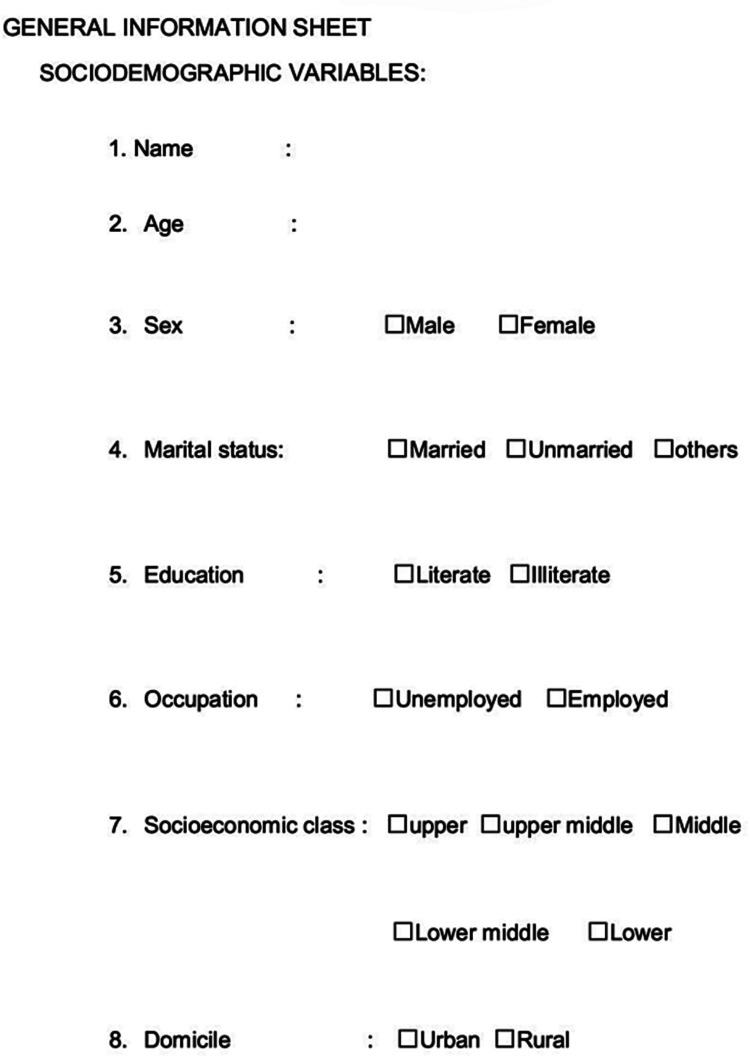
Self-designed form - page 1.

**Figure 7:**
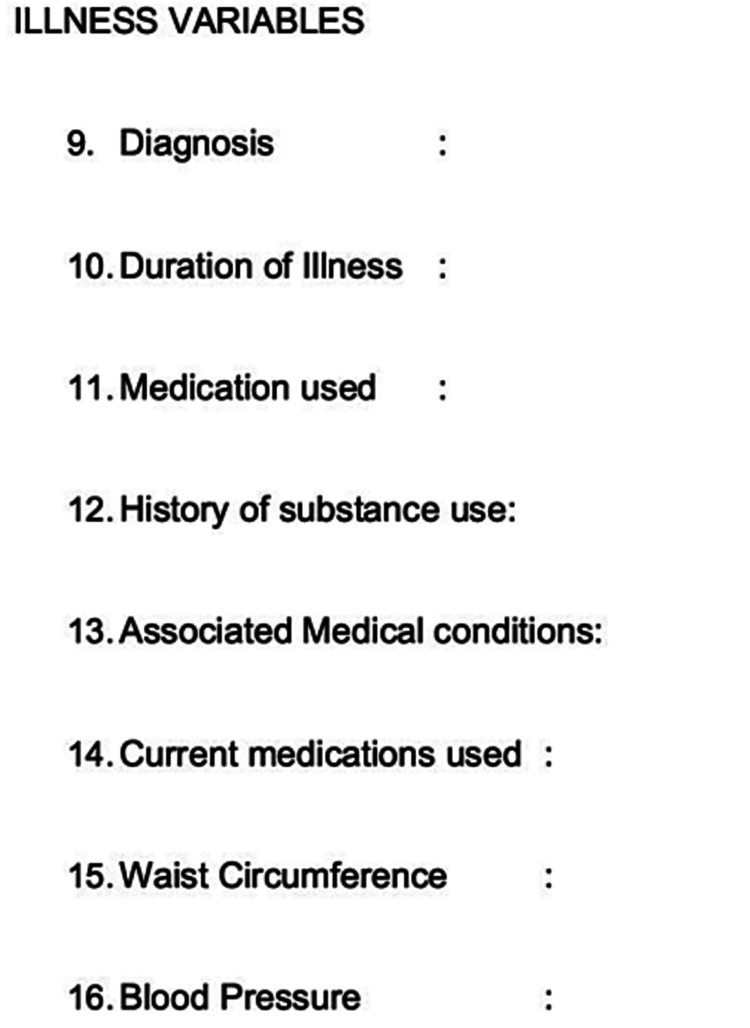
Self-designed form - page 2.

**Figure 8:**
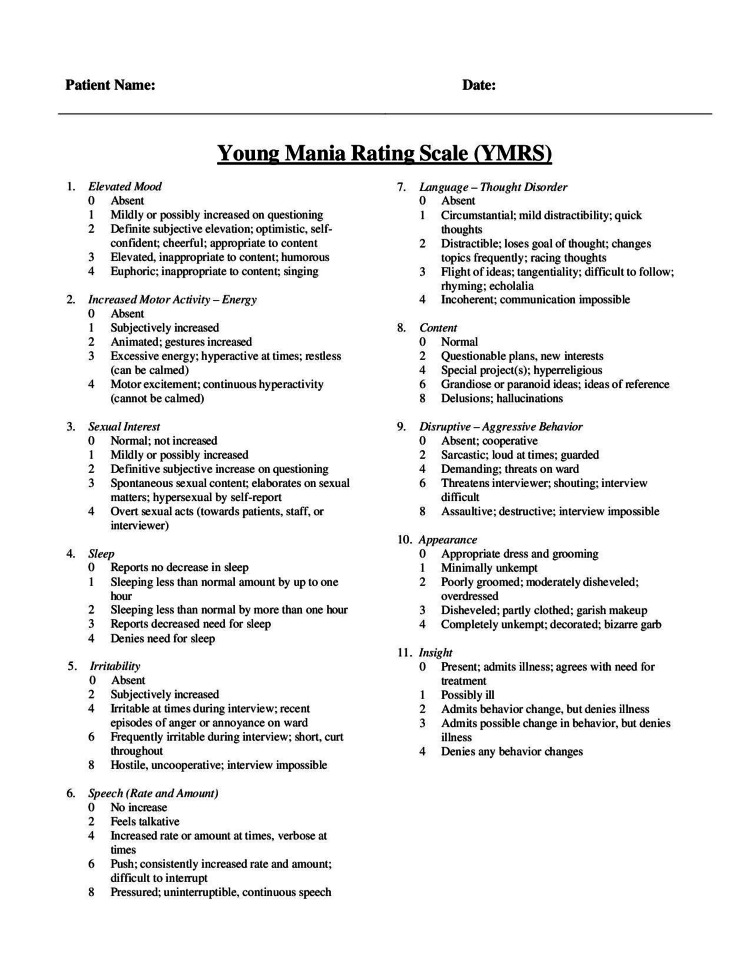
Young Mania Rating Scale (YMRS).

**Figure 9:**
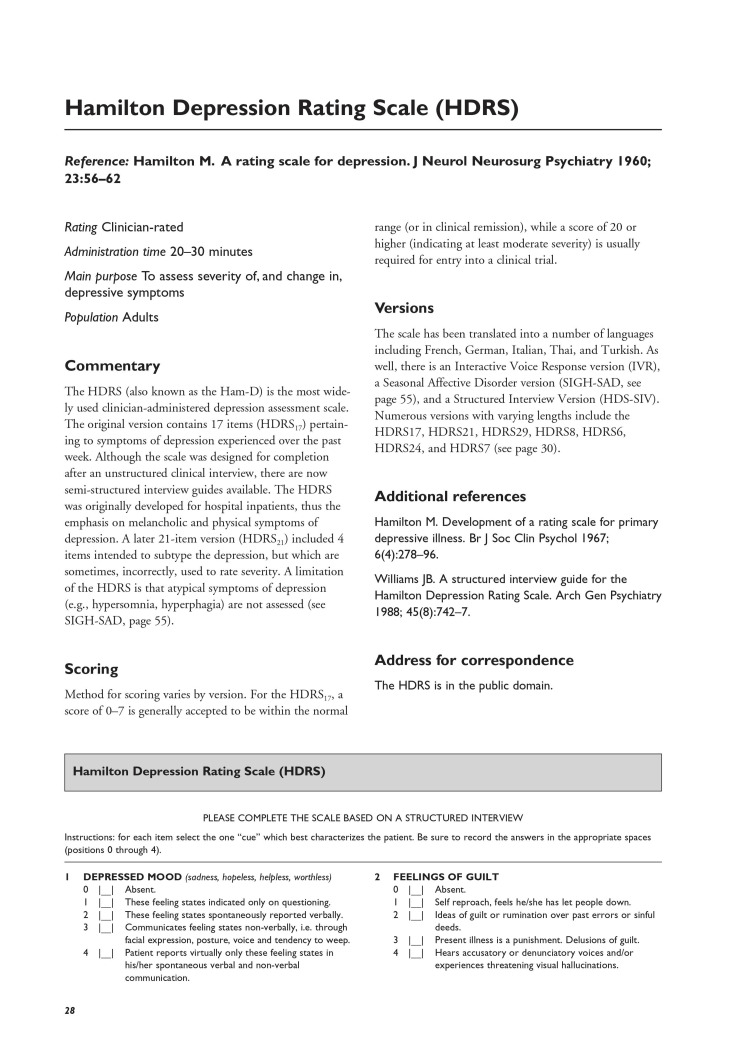
Hamilton Depression Rating Scale (HAM-D) - page 1.

**Figure 10:**
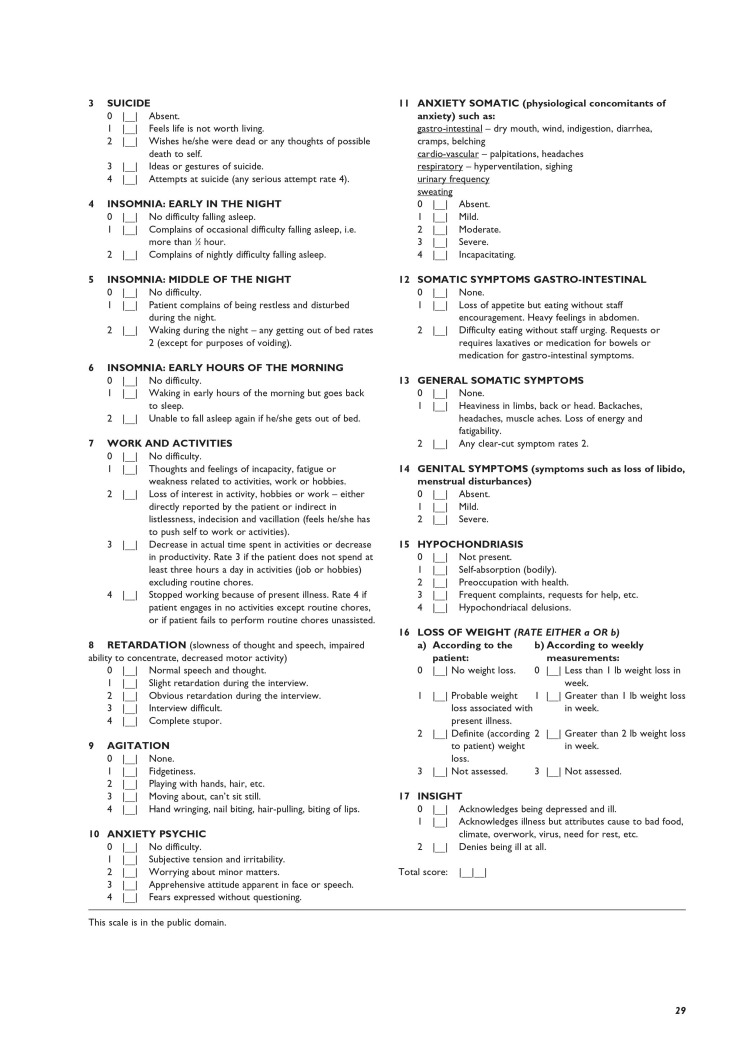
Hamilton Depression Rating Scale (HAM-D) - page 2.

A significant and positive relationship was observed between the severity of illness in the BPAD group, as measured by YMRS score, and serum uric acid levels. Specifically, the correlation coefficient was 0.698, indicating a strong association between the two variables (p < 0.01), as illustrated in Figure 2. On the other hand, the relationship between the severity of illness in the BPAD-depression group, as measured by HAM-D scores, and serum uric acid levels was found to be moderately positive (r = 0.574, p < 0.01), which is illustrated in Figure 3. In the MDD group, a weaker positive correlation was observed between the severity of illness, as measured by the HAM-D score, and serum uric acid levels, with a correlation coefficient of 0.289 and a significance level of less than 0.01, as illustrated in Figure 4.

In the BPAD-mania group, 37 patients (28.5%) were taking lithium carbonate, 26 patients (20%) were taking sodium valproate, and 15 patients (11.5%) were taking carbamazepine (Table 3). A statistically significant reduction in blood uric acid levels was observed in all three treatment groups: lithium carbonate, sodium valproate, and carbamazepine. Specifically, the mean change in blood uric acid levels was 2.0±0.4 mg/dL in the lithium carbonate group, 2.1±0.5 mg/dL in the sodium valproate group, and 2.3±0.7 mg/dL in the carbamazepine group. However, a statistical analysis using ANOVA did not show any significant differences between the three groups.

**Table 3:** Changes in blood uric acid levels before and after treatment by medication type.

Type of medication	Difference in blood uric acid before and after treatment			P-value
	N	Mean	SD	
Lithium carbonate	37	1.98	0.41	0.113
Sodium valproate	26	2.09	0.52	
Carbamazepine	15	2.32	0.74	

## Discussion

Prior to treatment, a significant difference was noted statistically in the mean blood uric acid levels among the three groups (BPAD-mania, BPAD-depression, and MDD), suggesting that the groups exhibited distinct variations in uric acid levels before receiving treatment. Notably, individuals with BPAD-mania displayed significantly elevated blood uric acid levels compared to the other groups, aligning with the results of numerous previous studies that have reported similar findings [5-9,19,20]. Interestingly, the individuals with BPAD-depression demonstrated a significant elevation in blood uric acid levels compared to those with MDD, which is consistent with previous research findings [21-23]. Given these findings and those from the literature, it appears reasonable to hypothesize that uric acid levels may serve as a biomarker for distinguishing between BPAD and unipolar depression.

Following treatment, the blood uric acid levels in patients with bipolar mania decreased by approximately 40%. A statistical analysis showed a significant difference in blood uric acid levels before and after treatment in each of the three groups, with a greater difference observed in the BPAD group compared to the MDD group. This outcome is consistent with the findings of De Berardis et al. [24], who reached similar conclusions in their earlier study. According to a survey by Keshavarz et al. [19], patients with MDD exhibited elevated blood uric acid levels during the remission phase, as compared to the acute phase, which may be related to chronic antidepressant use.

This study identified a significant correlation between blood uric acid levels and the severity of manic episodes in individuals with BPAD, as assessed by the YMRS. A notable correlation was also observed in the bipolar BPAD group, assessed using the YMRS score (r-value: 0.698). In contrast, a weaker association was observed in the MDD group, as evaluated by the HAM-D, with an r-value of 0.289. This observation is in line with previous research by De Berardis [24], which identified a significant positive correlation between blood uric acid levels and illness severity across all three study groups. In contrast, a separate study [25] discovered a statistically significant positive link between serum uric acid levels and impulsivity, but not with YMRS scores. Keshavarz et al. [19] reported no correlation, while Kiran et al. [26] found a negative correlation. A study by Kesebir et al. [27] found a moderate link between uric acid levels and personality traits related to hyperthymia (positive outlook) and irritability. However, they also discovered an opposite relationship between uric acid levels and depressive symptoms, which was stronger in individuals with MDD. In other words, the study found that patients with higher uric acid levels were less likely to experience depressive symptoms, particularly in those with MDD.

This study identified a strong correlation between mood stabilizers (lithium carbonate or sodium valproate, or carbamazepine) and uric acid levels. This finding is consistent with the results of Anumonye et al. [28], who previously demonstrated that lithium compounds are associated with reduced uric acid levels and a uricosuric effect in individuals with mania. Another study has demonstrated that carbamazepine decreases uric acid levels, whereas valproate has the opposite effect, increasing uric acid levels. A recent study [29] found that patients who received a combination therapy of lithium and valproate experienced higher uric acid levels compared to those treated with lithium monotherapy or lithium combined with carbamazepine. In addition, this study also revealed a statistically significant positive correlation between uric acid levels and serum lithium levels, which is consistent with the current study's findings.

Interestingly, there is no significant difference based on the type of mood stabilizers administered to subjects and their impact on serum uric acid levels. To the best of our knowledge, there is a dearth of existing research that enables a direct comparison of current observations, thereby limiting the ability to make informed conclusions.

The elevated blood uric acid levels in patients with BPAD compared to those with other psychiatric disorders may contribute to the increased vulnerability of BPAD to recurrences. This is because the adenosine receptors, which limit cellular excitability by inhibiting neurotransmitter release, are sensitive to uric acid levels. As a result, the elevated uric acid levels in BPAD may disrupt the normal functioning of adenosine receptors, leading to increased neural excitability and a higher likelihood of relapse. Alternatively, the rise in blood uric acid levels may be a byproduct of the manic episode itself, rather than a pre-existing condition that triggers mania and could potentially perpetuate manic symptoms as a result of purinergic dysfunction [21]. Despite ongoing debate, the relationship between purinergic dysfunction, as evidenced by elevated serum uric acid levels, remains unclear: Does this phenomenon serve as a hallmark indicator of mania episodes or is it a stable characteristic inherent to individuals with BPAD? Our study controlled for the effects of metabolic and renal function factors, as well as co-occurring alcohol use disorder, to isolate the relationship between manic symptoms and elevated uric acid levels in individuals with BPAD. This investigation provides additional evidence for a distinct dysfunction of the purine system in individuals experiencing manic episodes [30].

A novel approach proposed by Machado-Vieira suggests that investigating blood uric acid levels as a screening test for BPAD-mania could enable earlier identification of individuals with BPAD who exhibit hyperuricemia, a condition associated with elevated blood uric acid levels. This group might benefit from targeted treatment, including the addition of allopurinol, a medication that has been shown to be effective in managing hyperuricemia. This approach may facilitate tailored treatment approaches that optimize clinical outcomes and reduce treatment-related side effects.

## Conclusions

This study has demonstrated a significant difference in blood uric acid levels between individuals with BPAD-mania, BPAD-depression, and MDD. Notably, all patient groups showed a decrease in blood uric acid levels following treatment, with those with BPAD experiencing a more substantial reduction compared to the MDD group. Moreover, a strong correlation between blood uric acid levels and symptom severity was observed in individuals with bipolar mania. The study's findings provide strong evidence that dysfunction in the purine system plays a crucial role in the development of BPAD, regardless of its chronicity or medication exposure. These results suggest a previously unknown mechanism underlying the pathophysiology of BPAD, offering new insights into the disease's complex nature.

The strength of this study lies in its comprehensive examination of serum uric acid levels in all groups, both before and after treatment, while controlling for a range of influential factors. What sets this study apart is its meticulous consideration of various variables that can impact serum uric acid levels, including metabolic syndrome, renal diseases, and psychoactive substance use. Furthermore, the study explores the correlation between serum uric acid levels and various clinical variables, providing a nuanced understanding of the relationship between these factors. This study has some limitations. For example, we could have gathered more comprehensive data on serum uric acid levels during the remission phase by conducting a longer follow-up assessment. Alternatively, we could have chosen to conduct the study with a larger sample size, which may have increased the generalizability of our results by allowing for more representative findings. Additionally, we were unable to establish a correlation between the antipsychotics used and the change in blood uric acid levels, as this was not feasible with our current methodology.
